# Uterine cancer classification from CT images using convolutional feature extraction and transformer-based self-attention

**DOI:** 10.3389/fmed.2026.1781499

**Published:** 2026-02-25

**Authors:** Eman Hussein Alshdaifat, Amer Mahmoud Sindiani, Salem Alhatamleh, Rami Malkawi, Rola Madain, Rawan Eimad Almahmoud, Bara'a Al-Smadi, Asma'a Mohammad Al-Mnayyis, Mohammad Amin, Alaa Abd-alrazaq

**Affiliations:** 1 Department of Obstetrics and Gynecology, Faculty of Medicine, Yarmouk University, Irbid, Jordan; 2 Department of Obstetrics and Gynecology, Faculty of Medicine, Jordan University of Science and Technology, Irbid, Jordan; 3 Computer Science Department, Faculty of Information Technology and Computer Sciences, Yarmouk University, Irbid, Jordan; 4 Department of Information Systems, Faculty of Information Technology and Computer Science, Yarmouk University, Irbid, Jordan; 5 Department of Internal Medicine, College of Medicine, Yarmouk University, Irbid, Jordan; 6 AI Center for Precision Health, Weill Cornell Medicine-Qatar, Qatar Foundation, Doha, Qatar

**Keywords:** classification, CT images, deep learning, diagnosis, real dataset, uterine cancer

## Abstract

**Background:**

Accurate and early diagnosis of uterine cancer from computed tomography images remains a challenging task due to the complexity of anatomical structures and the subtle visual differences between normal, benign, and malignant uterine tissues. Traditional diagnostic approaches and conventional deep learning models often fail to effectively capture both local and global image characteristics.

**Objective:**

This study aims to develop and validate a novel hybrid deep learning framework that integrates convolutional feature extraction with transformer-based global attention mechanisms to improve the accuracy and robustness of uterine cancer classification from computed tomography images.

**Methods:**

In the proposed framework, DenseNet121 is employed as a convolutional neural network feature extractor, while a transformer encoder is utilized to model long-range contextual dependencies through multi-head self-attention. DenseNet121 captures discriminative local features from computed tomography images, which are subsequently processed by the transformer to enhance global feature representation. The performance of the proposed model is evaluated using the KAUH uterine cancer computed tomography dataset, which includes three classes: normal, benign, and malignant. The proposed approach is compared with several state-of-the-art deep learning models, including VGG16, VGG19, MobileNetV2, ResNet50, and DenseNet121.

**Results:**

Experimental results demonstrate that the proposed hybrid model outperforms the comparative models. It achieves an accuracy of 87.44%, sensitivity of 87.13%, specificity of 95.20%, an F1 score of 87.17%, and an area under the receiver operating characteristic curve of 99.41%.

**Conclusion:**

The results confirm the effectiveness of integrating convolutional neural networks with transformer-based self-attention mechanisms for significantly improving uterine cancer classification from computed tomography images. The proposed model shows strong potential as a computer-aided decision-support tool for radiologists to assist in the detection of uterine cancer and may be extended to various real-world clinical applications.

## 1 Introduction

Uterine tumors, which include both benign and malignant tumors, create a substantial healthcare challenge because of their high occurrence rates. Uterine cancer constitutes the most frequently occurring malignancy in women throughout the United States, which results in a 3.4% lifetime cancer risk (1). The sixth most prevalent cancer in 2012 (2) affects breast cancer as the most common cancer among women in the United States. The most common tumor type that affects women is leiomyoma (3). The tumors develop during the female reproductive life along with the perimenopausal period, thus creating debilitating symptoms of hemorrhage, pelvic heaviness, and pain (4). The malignant varieties of uterine cancers include endometrial cancer along with uterine sarcomas, which have a poor prognosis despite being a rare type of cancer.

Uterine cancers develop among women who experience advanced age and reach menopause later and who show hormonal imbalances and who have never given birth (5). The relationship between genetic pre-dispositions such as Lynch syndrome and obesity, which causes increased estrogen levels, which can lead to tumor development, serves as an additional risk factor (6). Researching the risk factors associated with uterine cancer proves necessary because of rising cancer rates and growing death rates which reached 1.1 increase from 1999 to 2016 with black women experiencing higher rates than white women (7). The medical establishment achieves its mission to decrease patient death rates while delivering better patient care through the practice of diagnosing diseases at their earliest stages and providing prompt medical intervention.

Deep learning algorithms have been applied for the evaluation and interpretation of images obtained from CT scans with the objective of improving diagnostic accuracy. For instance, they have been applied to help in distinguishing between benign and malignant uterine smooth muscle tumors (8). Deep learning enables automatic tumor segmentation which has become a vital component of treatment planning. The ability of deep learning in improving the diagnostic accuracy of CT images has been proven in studies involving ovarian cancer (9), which further supports its application for the evaluation of uterine smooth muscle tumors. The deep learning method for staging endometrial cancer using MRI images has produced positive results according to the research findings (10). The applications face multiple difficulties because of dataset discrepancies which create the main obstacles. Future studies should develop models which exhibit reduced variations while including multimodal imaging elements according to (11).

Although there are significant advances in the employment of deep learning techniques in medical image analysis, there are still challenges to be encountered in diagnosing uterine cancer from computed tomography images. Most previous works have harnessed CNN architectures that are mostly designed and trained for extracting local features, with limited representation of the long-range contextual relations within the image. Thus far, only a limited number of studies have focused on the classification of uterine cancer CT images into normal, benign, and malignant categories with high accuracy, using realistic datasets from clinical settings. In general, the following contributions can be summarized as part of the research study in uterine cancer diagnosis using artificial intelligence (AI):

- A novel hybrid deep learning framework is proposed, combining the DenseNet121 network for local feature extraction with a Transformer Encoder for capturing long-range contextual dependencies using a multi-headed self-attention mechanism.
- The accuracy of classifying CT images of uterine cancer into three categories (normal, benign, and malignant) is improved, surpassing the performance of traditional deep learning models such as VGG16, VGG19, MobileNetV2, ResNet50, and DenseNet121.
- The proposed model's effectiveness is experimentally verified using a real clinical dataset (KAUH-UCCTD), enhancing the reliability of the results and their generalizability for real-world medical applications.
- Outstanding performance is demonstrated across several evaluation metrics, including accuracy, sensitivity, specificity, F1 score, and AUC curve, showing the model's strength in differentiating between various conditions and reducing diagnostic errors.
- Presenting a promising model as a clinical decision support system that can assist radiologists in the early and accurate detection of uterine cancer, with the potential to be developed and expanded for use in actual clinical settings.

The subsequent sections are arranged as follows: a thorough analysis of the pertinent research on uterine diseases is given in Section 2. In Section 3, the dataset is described, the applied methodology is described, and the structure of the suggested model is explained in depth. The experimental outcomes of the suggested model are shown in Section 4. The scalability and comprehensiveness of the suggested model are covered in Section 5, and the results are summarized and future research opportunities are outlined in Section 6.

## 2 Related work

Deep learning models used for many types of medical imaging, including computed tomography, magnetic resonance imaging, and histology imaging, have progressively helped diagnose uterine cancer and classify benign and malignant tumors. The most recent findings from studies on using AI for uterine cancer classification are presented in this section.

The study presented in (12) introduced a modified YOLOv5 framework for the classification of uterine abnormalities based on ultrasound images. The proposed study focused on the redesigning of the head module of the original YOLOv5 architecture, the addition of global attention mechanism (GAM), and the use of a modified activation function and ResNeXt-based CSP blocks to create a new backbone to capture the very subtle features from the noisy ultrasound images more effectively. A dataset of 3,026 clinically-acquired ultrasound images was used for model training and validation, and the images were labeled into the classes: cervical cyst, uterine fibroid, and normal. Experimental results showed that the proposed method achieved an accuracy of 80.5%, indicating the effectiveness of architectural enhancements and attention processes in enhancing the classification of uterine abnormalities based on ultrasound.

In another research work (13), the goal is to offer an objective evaluation regarding the aspects influencing the success of UFE and to promote the use of an interpretable machine learning (ML) approach to aid in decision-making in the clinical setting via features extracted from pre-operative MRI. A carefully filtered dataset with 74 patients and 311 fibroids was created, and they used Deep Set Networks, which allow for permutation-equivariant aggregation of features over the collection of fibroids. Accuracy values of 81–88% (AUC = 0.81–0.87) were measured in individual symptoms for the proposed models, as well as 75% (AUC = 0.74) for the overall clinical success, whereas at the fibroid level, the prediction task by ensemble tree-based techniques resulted in an accuracy of 76% max.

Another study (14) presented the role of AI in the diagnosis and treatment of uterine fibroids and uterine sarcomas, covering studies published between 2019 and March 2025. The research examined various AI techniques which included radiomics machine learning and deep neural network systems that worked with ultrasound and MRI medical images. The methods successfully distinguished between benign leiomyomas and malignant leiomyosarcomas which helped doctors develop treatment plans and improved results from minimally invasive procedures including HIFU and uterine artery embolization. AI systems demonstrated better performance than expert radiologists in most cases according to the results while multiple studies reached diagnostic accuracy levels above 0.85 AUC scores.

In the study (15), a framework was proposed for surgical decision-making in uterine fibroid management through integrating female sex hormone levels with fibroid characteristics. The study included 618 women diagnosed with uterine fibroids (UFs) from a multicenter hospital, of whom 238 underwent surgery. Multiple supervised ML algorithms, such as support vector machine (SVM), decision tree (DT), random forest (RF), logistic regression (LR), and k-nearest neighbors (KNN), with 126 different input combinations derived from hormonal markers (FSH, LH, E2, PRL, and AMH) and morphological variables. The RF model achieved the highest accuracy of 91% and an AUC of 0.88 using LH, FSH, E2, and AMH. The model showed a high level of clinical concordance through external validation using 20 independent cases, achieving a 90% agreement rate with a blinded gynecologist.

As per the study (16), a multicenter deep learning-based framework for automatically outlining clinical target volumes (CTV) and planning target volumes (PTV) in radiotherapy for uterine cancers. The research employed the self-configuring nnU-Net architecture to solve manual contouring challenges which included observer differences and institutional result variations and heavy workload demands. The researchers collected a dataset which contained 602 contrast-enhanced CT scans that included cervical and endometrial cancer cases from multiple medical centers. The researchers conducted a detailed evaluation of three nnU-Net configurations which included 2D slice-level and 3D full-resolution and 3D cascaded models through testing with Dice Similarity Coefficient (DSC) and Average Surface Distance (ASD) and Hausdorff Distance (HD95). The 3D full-resolution nnU-Net provided the best overall results, with average DSC values of 83.42% for PTV and 81.23% for CTV in internal testing. Additionally, evaluations from experienced radiation oncologists showed that approximately 90% of the automatically created contours required no changes or only minor adjustments, demonstrating the clinical usefulness of the proposed method. While the study (17) introduced deep learning-based nnU-Net models, for automatic segmentation of uterine fibroids and their surrounding organs based on MRI images to support high-intensity focused ultrasound HIFU surgery planning. Used a retrospective dataset of 550 T2-weighted MR images. The evaluation showed that the proposed 3D nnU-Net significantly outperformed state-of-the-art methods such as HIFUNet, U-Net, R2U-Net, ConvUNeXt, and 2D nnU-Net, achieved DSC of 92.55% for the uterus, 89.63% for the endometrium, 90.45% for the urethral orifice, 97.75% for the bladder, 95.63% for fibroids, and 92.69% for the spine.

In a study (18) a novel deep learning-based 3D super-resolution DWI (SR-DWI) radiomics model was proposed for predicting the prognosis of high-intensity focused ultrasound (HIFU) ablation of uterine fibroids. Radiomics features were extracted from manually segmented fibroid regions and subsequently reduced using *t*-test, Pearson's correlation, and the LASSO regression algorithm. machine learning classifiers, including SVM, RF, and LightGBM, were trained and validated on multicenter datasets with both internal and external testing cohorts. All DWI radiomics models showed superior AUC, according to experimental results. The best-performing HR-DWI model (SVM) achieved an AUC of approximately 0.805 in internal testing, whereas the SR-DWI–based models demonstrated better performance, with AUC values of 0.876 in internal testing and 0.800 in external validation, indicating a statistically significant improvement over HR-DWI (*P* < 0.05). The findings show the potential of combining deep learning-based super-resolution imaging with radiomics. This approach can improve pre-operative prognostic assessment of eligible candidates for HIFU therapy.

According to the study (19) an image processing-based solution was proposed for the diagnosis of cervical cancer from uterine cervix images using transfer learning architectures to reduce workload and assist experts by leveraging deep convolutional neural networks. Histogram equalization was applied to enhance image contrast before classification, while to suppress noise, Gaussian filtering was used. Many transfer learning models, including AlexNet, MobileNetV2, DenseNet201, ResNet50, VGG19, and Xception, were systematically evaluated using a 10-fold cross-validation strategy on the Herlev pap-smear dataset. The experimental results showed that the VGG19 model achieved the best performance, with an accuracy of 98.26%, outperforming other models on the same dataset.

Another study (20) proposed a machine-learning-based radiomics framework using pre-operative contrast-enhanced computed tomography (CECT) images to differentiate uterine leiomyomas from leiomyosarcomas. After standardized pre-processing, PyRadiomics was used to extract the radiomic features, and three methods (Boruta, LASSO, and RFE) were used to select features, with multiple classifiers such as GLM, RF, and SVM. The diagnostic performance of the proposed models reached strong results because they achieved test data AUC results between 0.78 and 0.97 which exceeded the performance of radiologists who achieved AUC results between 0.70 and 0.78 when the patient's clinical data were available. The study demonstrates how CT-based radiomics can function as a decision-support system during pre-operative evaluations. The EfficientNetB0 model achieved classification with an accuracy of 99% in the study (21) which used 1,990 ultrasound images to automatically classify uterine fibroids into two categories. The study employed an attention-based model which used attention mechanisms to guide the model towards important clinical areas while it neglected non-essential parts of the body. Table 1 presents a comparison of previous research on uterine diseases.

**Table 1:** Comparison of previous works on uterine diseases.

**Ref**	**Year**	**Methodology**	**Dataset**	**Results**	**Limitations**
(12)	2025	Improved YOLOv5 with enhanced ResNeXt backbone, and GAM attention for ultrasound image classification	3,026 ultrasound images	Accuracy: 80.5%	Single-center dataset
(13)	2025	Machine learning with deep set networks for patient-level prediction and fibroid-level prediction using MRI features	74 patients, 311 fibroids (MRI dataset)	Clinical outcome accuracy 75% (AUC = 0.74) symptom. prediction 81–88%. fibroid-level accuracy up to 76%	Single-center study small dataset
(14)	2025	AI methods applied to ultrasound, MRI, and HIFU workflows	Multiple prior studies (2019–2025), datasets range from small single-center cohorts to multicenter MRI/HIFU datasets	Achieved AUC values >0.85 for diagnosis and prognosis, strong performance	No new experimental validation
(15)	2025	Supervised ML models (RF, SVM, DT, LR, KNN) using female sex hormone parameters and fibroid features for surgical decision support	618 uterine fibroid patients from three hospitals	Best RF model 91% accuracy 90% agreement with a blinded gynecologist	No model calibration analysis
(16)	2025	nnU-Net–based deep learning (2D slice-level, 3D full-resolution, and 3D cascaded)	602 multicenter CT scans (cervical and endometrial cancers)	Best DSC: 83.42% (PTV), 81.23% (CTV), ~90% contours clinically acceptable	High computational cost for 3D models. CT-only data
(17)	2024	3D nnU-Net deep learning for multi-organ MRI segmentation and 3D reconstruction for HIFU planning	550 retrospective T2-weighted MRI scans	Significantly outperformed HIFUNet, U-Net, R2U-Net, ConvUNeXt, and 2D nnU-Net DSC = 95.63% (fibroids), 92.55% (uterus)	Single-center retrospective dataset
(18)	2024	DL-based 3D SR-DWI radiomics + ML (SVM, RF, LightGBM)	Multicenter MRI DWI datasets (360 patients, internal and external validation)	SR-DWI achieved AUC 0.876 (internal) and 0.800 (external), outperforming radiologists	Manual segmentation Limited clinical features included
(19)	2024	Image pre-processing (histogram equalization + Gaussian filter) + transfer learning CNNs (AlexNet, DenseNet201, MobileNetV2, ResNet50, Xception, VGG19)	Herlev Pap-smear	Accuracy: 98.26% f1-measure: 0.9671 specificity: 0.9896 sensitivity: 0.9631 precision: 0.9711 MCC: 0.9552	Limited to a single public dataset, binary classification only
(20)	2024	CT-based radiomics with ML (GLM, RF, SVM) and LASSO, Boruta, RFE	65 patients (30 leiomyosarcoma, 35 leiomyoma)	AUC from 0.78 to 0.97	Small sample size single-center study
(21)	2024	Attention-based fine-tuned EfficientNetB0 for ultrasound image classification	1,990 Ultrasound images	Accuracy = 99%	Single dataset

Hybrid deep learning systems development has demonstrated potential to improve medical imaging techniques and cancer detection methods. Alswilem and Pacal (22) conducted a comprehensive comparative study which assessed both computational efficiency and diagnostic accuracy of deep learning models used for automated breast cancer detection in ultrasound imaging. The study found that RexNet-200 achieved optimal performance with minimal computational resources which included 13.81 million parameters and 3.05 GFLOPs while maintaining 95 percent accuracy. The study demonstrates how model complexity needs to achieve a specific level which medical professionals need for their work throughout our DenseNet121-Transformer. Demirtaş Alpsalaz et al. (23) colleagues created a hybrid model which combines EfficientNet-B3 with Vision Transformer to detect colon cancer through their attention fusion mechanism and the system reached 96.2 percent accuracy while achieving an MCC score of 0.961. Their work demonstrated that attention-based fusion effectively harmonizes local texture features extracted by CNNs with global contextual dependencies captured by transformers, directly supporting our implementation of multi-head self-attention for feature integration. Çakmak (24) examined various machine learning techniques to improve the diagnostic process of hematological disorders and achieved 98.38 percent accuracy with LightGBM while showing that selecting features and optimizing models represent crucial components for building clinical decision support systems. Çakmak and Pacal (25) performed a study that compared four CNN architectures (ResNet50, DenseNet169, InceptionV3, InceptionV4) to classify breast ultrasound images and found that InceptionV3 achieved the best results with 96.67% accuracy and 96.55% precision. Their findings demonstrate that densely connected architectures together with multi-scale feature extraction systems work effectively which serves as our basis for selecting DenseNet121 to function as our convolutional current architecture. Alpsalaz et al. (26) created a deep learning model based on MaxViT which they used to classify Alzheimer's disease through MRI scans and achieved 99.60% accuracy by implementing transfer learning together with multi-axis attention mechanisms. This research shows how vision transformers successfully capture medical image details through their ability to detect both small and large image features which supports our choice to use transformer-based self-attention in our uterine cancer classification system.

Recent studies show that deep learning techniques successfully analyze medical images across different clinical settings. The medical field uses convolutional neural network approaches to detect and classify tumors which result in accurate brain tumor diagnosis through magnetic resonance imaging (27). The advanced object detection frameworks which include YOLO-based models successfully detect abdominal diseases because they use advanced feature extraction and pre-processing methods to handle complicated anatomical areas (28). The research demonstrates that data pre-processing and imbalance handling methods serve as essential elements which enhance machine learning models accuracy for medical diagnostic purposes (29). The research demonstrates that hybrid CNN-Transformer architectures with attention mechanisms work effectively in medical imaging applications because they provide strong theoretical and empirical foundations which support our proposed methodology.

## 3 Methods

### 3.1 Study design

**Figure 1:**
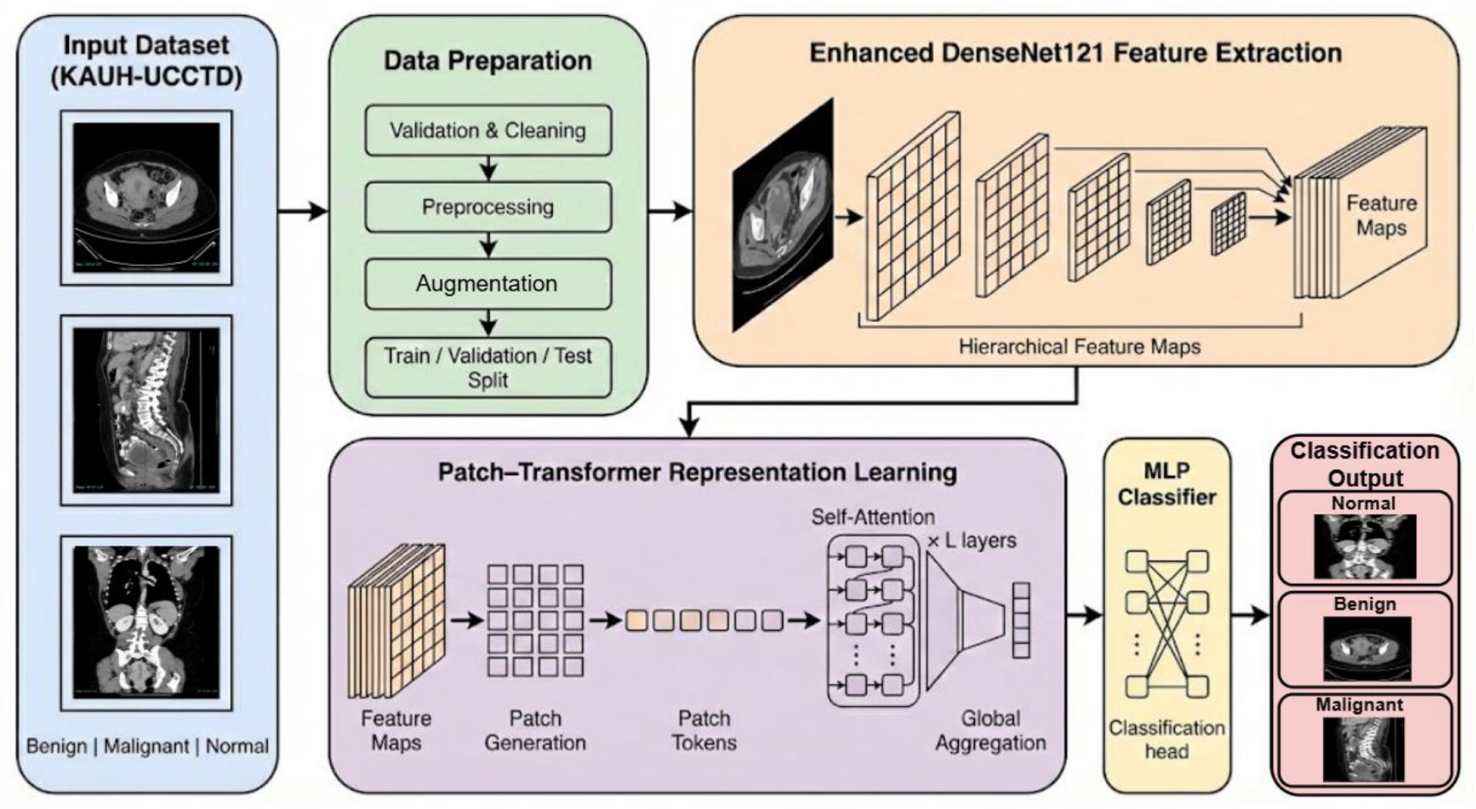
Overall architecture of the proposed DenseNet121-transformer hybrid model for uterine classification.

This study proposes a hybrid deep learning framework for the classification of uterine CT images from the King Abdullah University Hospital Uterine Cancer CT Dataset (KAUH-UCCTD) into three diagnostic classes: Benign, Malignant, and Normal. The methodology follows a systematic process that begins with dataset verification and controlled data partitioning, followed by standard image pre-processing and training-time data augmentation to enhance model robustness and generalization. As shown in Figure 1, an already-trained DenseNet121 is used as the feature-extraction backbone and is further improved by adding a patch embedding stage that turns the convolutional feature maps into a sequence of tokens, which are then fed into the stacked Transformer encoder layers where the long-range contextual dependencies are captured through self-attention. The resulting global representation is combined and sent to an MLP classification head to produce the final predictions, and the whole model is trained using a multi-class cross-entropy objective in an end-to-end manner.

### 3.2 Data collection from a hospital (KAUH)

**Figure 2:**
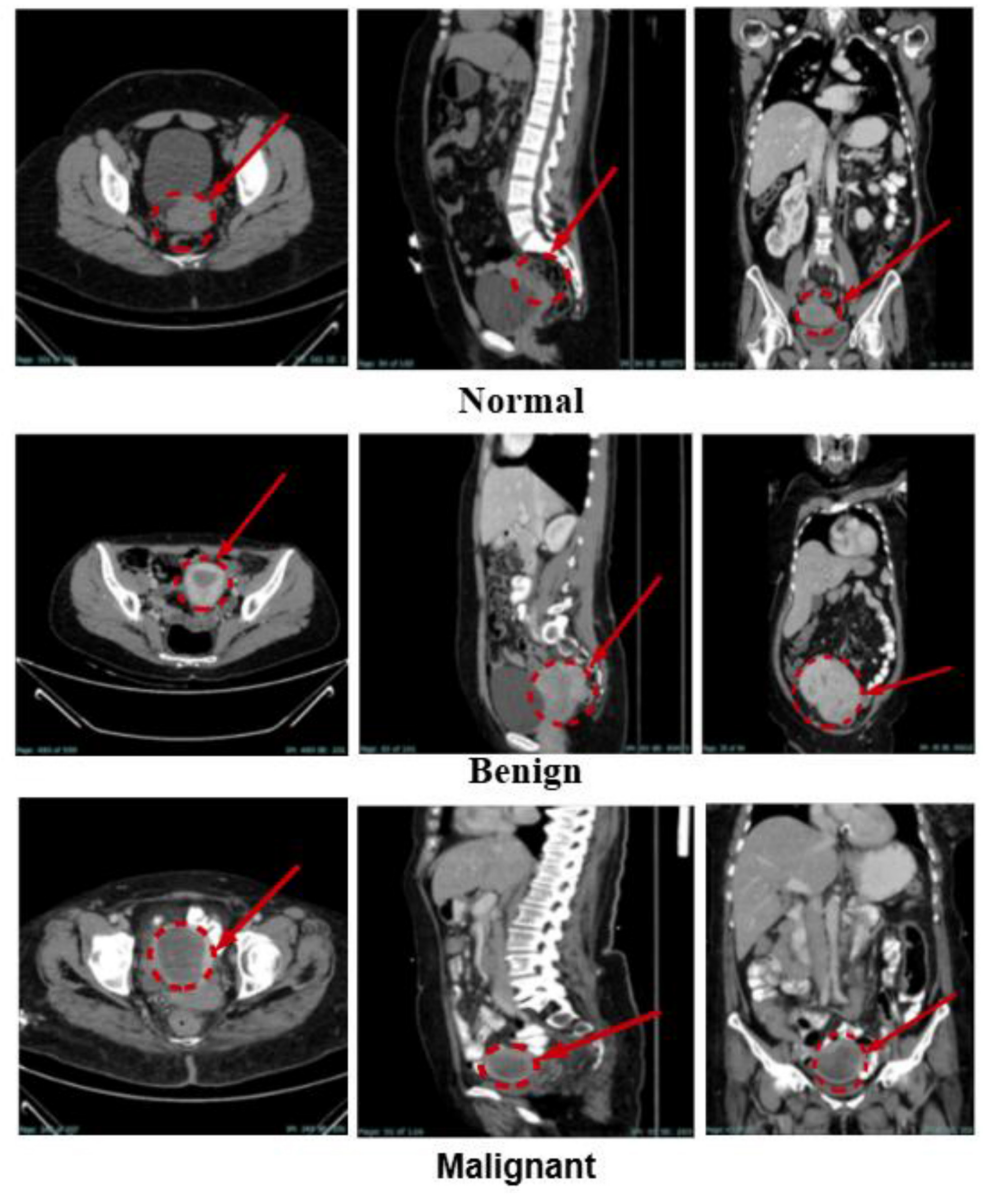
An example from the image dataset (KAUH-UCCTD).

In this study, KAUH-UCCTD was used. The Jordan University of Science and Technology's King Abdullah University Hospital provided the dataset, which contained 2,870 images from 600 women between the ages of 22 and 80. Numerous image slices were taken for each of the multiple imaging procedures that many of these patients had. The collection consists of images from patients who were retrospectively diagnosed by radiologists between early 2019 and late June 2024. Beginning in June and concluding in November 2024, the data collection process took 6 months. The photos were gathered, assessed, and classified by the hospital doctors before being filed. Three CT scan slices sagittal, coronal, and axial views are included in the collection. The pictures are downloaded in JPG format and saved on a 64-channel Philips Brilliance CT scanner. Table 2 displays the quantity and distribution of photos in each dataset category, while Figure 2 displays an example from every category.

**Table 2:** The Number and distribution of images in each KAUH-UCCTD category.

**Case**	**Number of images**
Normal	502
Benign	1,513
Malignant	855
**Total**	**2,870**

The bold value represents the total number of images across all categories.

To avoid potential data leakage, the dataset was split on a patient-level basis rather than a slice-level basis. All computed tomography slices belonging to the same patient were assigned exclusively to either the training, validation, or test set. This ensures that no overlapping patient information exists across different subsets and provides a fair and unbiased evaluation of the model's generalization performance.

### 3.3 Data pre-processing and augmentation

All CT images pass through a common pre-processing method unification to minimize variability and to standardize the input representations prior to training (30). The first step of the process is to resize the image spatially and then convert it into a three-channel format that matches the pre-trained feature extraction backbone. Furthermore, to aid the optimization process and to make the distribution of intensities similar among the samples, pixel values are normalized for each channel independently. This is normalization process equation.


$$\begin{array}{l} x_{norm}=\frac{x-\mu }{\sigma } \end{array}$$ (1)

Where *x* is the original input image tensor, μ is the channel-wise mean vector, and **σ** is the corresponding standard deviation vector. This transformation guarantees that the input features are centered and scaled to ranges that are comparable prior to inference by the model. To solve the problem of data scarcity and inter-class imbalance, the usual data augmentation techniques were performed on the training subset. Let us call the original training set dataset, where *x*_*i*_ is an image sample and *y*_*i*_ its corresponding class label. An augmentation operator A(·) is used to create more transformed samples:


$$D_{aug}=\{(A(x_{i}),y_{i})\}$$ (2)

The augmentation process increases intra-class variability while preserving semantic class identity, and is used to equalize the number of samples across classes, resulting in a balanced training distribution (31). Following pre-processing and augmentation, the dataset is partitioned into three mutually exclusive subsets: a training set $D_{train}=80\%$, a validation set $D_{val}=10\%$, and a test set $D_{test}=10\%$. The training subset is used for parameter optimization, the validation subset guides model selection and convergence control, and the test subset is reserved for final performance evaluation, ensuring an unbiased assessment of the proposed framework.

### 3.4 Model architecture and design

#### 3.4.1 Enhanced DenseNet121 feature extraction

The DenseNet121 architecture was adopted as the feature extraction backbone, as its dense connectivity enhances information flow across layers and supports robust representation learning. The patterns allow for efficient feature reuse and also help the gradient to propagate easily through the deep layers. Let **I** ∈ ℝ^*H*×*W*×*C*^ denote a pre-processed CT image that is fed into the network (32). The input image is processed through a series of densely connected convolutional blocks, where each block progressively transforms the input into higher-level feature representations. The resulting feature map is denoted as $\text{F}\in {\mathbb{R}}^{H_{f}\times W_{f}\times C_{f}}$, and the overall transformation performed by the DenseNet121 backbone is represented by Φ(·). Unlike the standard DenseNet121 configuration, which applies global pooling followed by direct classification using high-level features, the proposed framework modifies this pipeline by further refining the extracted feature map *F* to better support subsequent global modeling. Specifically, a normalization operation is applied to stabilize the feature distribution (33), followed by a non-linear activation function to enhance representational capacity.


$$\begin{array}{l} F=\text{Φ}\left(\text{I}\right) \end{array}$$ (3)

$$F^{'}=\sigma (BN(F))$$ (4)

where *BN*(·) denotes batch normalization and σ(·) stands for rectified linear activation function. The enhancement phase promotes feature consistency and prepares the representation for downstream tokenization. The enhanced feature map **F**′ still contains all the rich spatial and semantic information while exhibiting reduced redundancy due to dense feature reuse. The architecture proposed here overcomes the local limitation of pure CNN-based classifiers by taking the convolutional output right before any global pooling operation and thus allowing smooth integration with the following patch embedding and Transformer encoder modules. As a result, DenseNet121 serves not only as a local feature extractor but also as a robust foundation for capturing both fine-grained anatomical details and high-level contextual patterns present in uterine CT images.

#### 3.4.2 Patch-transformer representation learning

Following convolutional feature extraction, the enhanced feature map produced by the DenseNet121 backbone is transformed into a sequence-based representation suitable for attention-based modeling. Let $F^{'}\in {\mathbb{R}}^{H_{f}\times W_{f}\times C_{f}}$denote the refined convolutional feature map obtained from the previous stage. To bridge the gap between convolutional and Transformer-based processing (34), a patch embedding operation is applied to partition **F**′ into a set of non-overlapping patches. This operation can be expressed as equation (35).


$$P=\Psi (F^{'})$$ (5)

In this case, the symbol Ψ(·) stands for a learnable patch embedding function that is executed through a convolutional projection, while $\text{P}\in {\mathbb{R}}^{N_{p}\times D}\;$illustrates the final patch token sequence (36). In this situation, *N*_*p*_ refers to the complete number of patches that have been broken down from the feature map, whereas *D* is the size of the corresponding token embedding dimension. The tokenization method used keeps the localized spatial information intact while allowing its sequential modeling. Moreover, since the Transformer architecture is characterized by its permutation-invariance, it becomes mandatory to inject explicit positional information into the system in order to maintain the spatial order among the tokens. Thus, a set of learnable positional embeddings ${\text{E}}_{pos}\in {\mathbb{R}}^{N_{p}\times D}$ is appended to the patch token sequence.


$$\begin{array}{l} {\text{X}}_{0}=\text{P}+{\text{E}}_{pos} \end{array}$$ (6)

where **X**_0_ stands for the position-aware token representation. By carrying out this step, the model is made capable of telling the difference between the patches that come from different spatial locations in the original CT image. The position-modulated tokens are next fed into a series of Transformer encoder layers to discover long-range dependencies and global contextual relationships. Each Transformer encoder layer consists of a multi-head self-attention (MHSA) mechanism followed by a feed-forward multilayer perceptron (MLP), both wrapped with residual connections and layer normalization (37). Given an input token sequence **X**, the self-attention operation is defined as an equation.


$$\begin{array}{l} Attention\;\left(\text{Q},\text{K},\text{V}\right)=Softmax\left(\frac{\text{Q}{\text{K}}^{T}}{\sqrt{d_{k}}}\right)\text{V} \end{array}$$ (7)

In the above-mentioned process, **Q**, **K**, and**V** signify the query, key, and value matrices generated from **X**, respectively, whereas *d*_*k*_ refers to the size of the individual attention head. The features of various attention heads get concatenated and then linearly projected to form the final output of MHSA (38). For making training stable and allowing gradient flow, residual learning is applied in every encoder block, giving rise to the following transformations.


Z=X+MHSA(LN(X) (8)

$$\begin{array}{l} \text{Y}=\text{Z}+MLP\left(LN\left(\text{Z}\right)\right) \end{array}$$ (9)

Where *LN*(·) is the layer normalization function and Y is the output from one of the Transformer encoder layers. When multiple layers are combined, the model can take advantage of local convolutional features as well as global contextual information all across the image. This representation learning approach based on Patch-Transformer not only surpasses the locality constraint of convolutional networks but also allows for drawing relations between far-off anatomical regions that capture fine and spatially distributed patterns in uterine CT images which is a necessity.

#### 3.4.3 Classification and training strategy

Following the Patch-Transformer learning of representation, the output token sequence $\text{Y}\in {\mathbb{R}}^{N_{p}\times D}$ carries the information of both local spatial features and global contextual relationships. A global aggregation operation is done across the token dimension in order to get a fixed-length representation that is suitable for classification. If **y**_*i*_ denote is the *i*-th token in the sequence, then the aggregated feature vector **g** ∈ ℝ^*D*^ is computed as follows.


$$\begin{array}{l} \text{g}=\frac{1}{N_{p}}\overset{N_{p}}{\underset{i=1}{\sum }}{\text{y}}_{i} \end{array}$$ (10)

This operation summarizes the overall image representation while preserving the discriminative information learned by the Transformer encoder. The resulting vector **g** is then passed to a MLP classification head (39), which serves as the decision-making component of the proposed framework. The MLP head consists of a sequence of fully connected layers with non-linear activation functions and regularization mechanisms, and produces a vector of class logits.


$$\begin{array}{l} \hat{\text{y}}=f_{cls}\left(\text{g}\right) \end{array}$$ (11)

where *f*_*cls*_(·) represents the mapping executed by the MLP head and $\hat{\text{y}}\in {\mathbb{R}}^{C}$ refers to the unnormalized prediction scores for Cdiagnostic classes. For the complete training of the network, a multi-class cross-entropy loss function is used. The loss is calculated between the true label vector *y* and the predicted class probabilities $\hat{\text{p}}$ obtained through a softmax transformation, and is given as:


$$\begin{array}{l} L=-\overset{C}{\underset{c=1}{\sum }}y_{c}\log\left({\hat{p}}_{c}\right),\hat{\text{p}}=softmax\left(\hat{\text{y}}\right) \end{array}$$ (12)

Where *y*_*c*_ and ${\hat{p}}_{c}$ represent the actual and estimated probabilities for class *c*, respectively. This aim pushes the model to be very sure about the right diagnostic category. A gradient-based adaptive optimizer is used for model optimization, and a validation-guided learning rate scheduling strategy dynamically controls the learning process. The situation in which the schedule tracks the validation loss and consequently modifies the learning rate leads to the stagnation of convergence process which in return makes training stable and evades the trap of the model getting stuck in poor local minima. Among the regularization techniques, dropout applied in the MLP head helps in significantly reducing overfitting and consequently improving the model's performance on unseen data (40). In general, this classification and training policy guarantees that the proposed architecture can successfully convert rich Patch-Transformer representations into trustworthy diagnostic predictions while at the same time being robust and reproducible across training runs.

### 3.5 Evaluation protocol

To ensure that the proposed model is assessed in a clinically meaningful manner, evaluation is not limited to a single aggregate score. Instead, a set of complementary metrics is reported to reflect different diagnostic risks and decision priorities encountered in uterine cancer screening and triage (41). Accuracy provides an overall estimate of correctness; however, in clinical practice it may hide critical failure modes when the number of normal cases differs from abnormal cases. Therefore, Precision is used to quantify how trustworthy a positive prediction is (i.e., when the model flags a case as malignant/abnormal, how often this alert is correct). This is directly relevant to reducing unnecessary follow-up imaging, biopsies, and patient anxiety associated with false-positive findings. Sensitivity (Recall) detects true disease cases and is often the most important standard in cancer treatment because if a tumor is missed, the treatment may be delayed, and negative results will follow. Oncology may thus rely on this criterion most heavily. Sensitivity is complemented by Specificity, which indicates how well the system correctly identifies non-disease cases. Specificity further supports clinic workflow efficiency by avoiding over-referral and lightening the load on both radiology and oncology services. The F1-score issue a single balanced indicator that together with precision and sensitivity, thus being especially useful when clinicians ask for one summary measure and yet wishing the reflection of both false-alarm and missed-case behaviors. Lastly, since clinical decision thresholds might differ from one hospital to another and depend on screening policies, a threshold-independent assessment is carried out by means of ROC analysis and AUC (42). By means of ROC curves, the trade-off between sensitivity and the false-positive rate is visualized as the decision threshold is varied while AUC captures the model's global discriminative power; combined they demonstrate whether the model can differentiate malignant from non-malignant patterns consistently across various operating points and risk tolerances. In case of a multi-class situation (Benign/Malignant/Normal), these metrics can be evaluated for each class and also simultaneously computed, allowing doctors to view class-wise reliability (e.g., malignant detection) instead of relying on an overall score only.


$$\begin{array}{l} Accuracy=\frac{TP+TN}{TP+TN+FP+FN} \end{array}$$ (13)

$$\begin{array}{l} Precision=\frac{TP}{TP+FP} \end{array}$$ (14)

$$\begin{array}{l} Sensitivity=\frac{TP}{TP+FN} \end{array}$$ (15)

$$\begin{array}{l} Specificity=\frac{TN}{TN+FP} \end{array}$$ (16)

$$\begin{array}{l} F1-Score=2\cdot \frac{Precision\cdot Sensitivity}{Precision+Sensitivity} \end{array}$$ (17)

## 4 Results

This section will go over the findings of the study's suggested model, which was tested using the KAUH-UCCTD uterine tumor datasets that were gathered from King Abdullah University Hospital in Jordan. Each model was trained using the same set of parameters: 50 epochs, learning rate of 0.0001, Adam activation function, batch size 16, image size (224,224), and class cross-entropy loss function. The dataset is divided into three subsets for this study: 10% for testing, 10% for validation, and 80% for training. Additionally, a Jupyter laptop and an RTX 3050 GPU were used to train the models locally.

**Figure 3:**
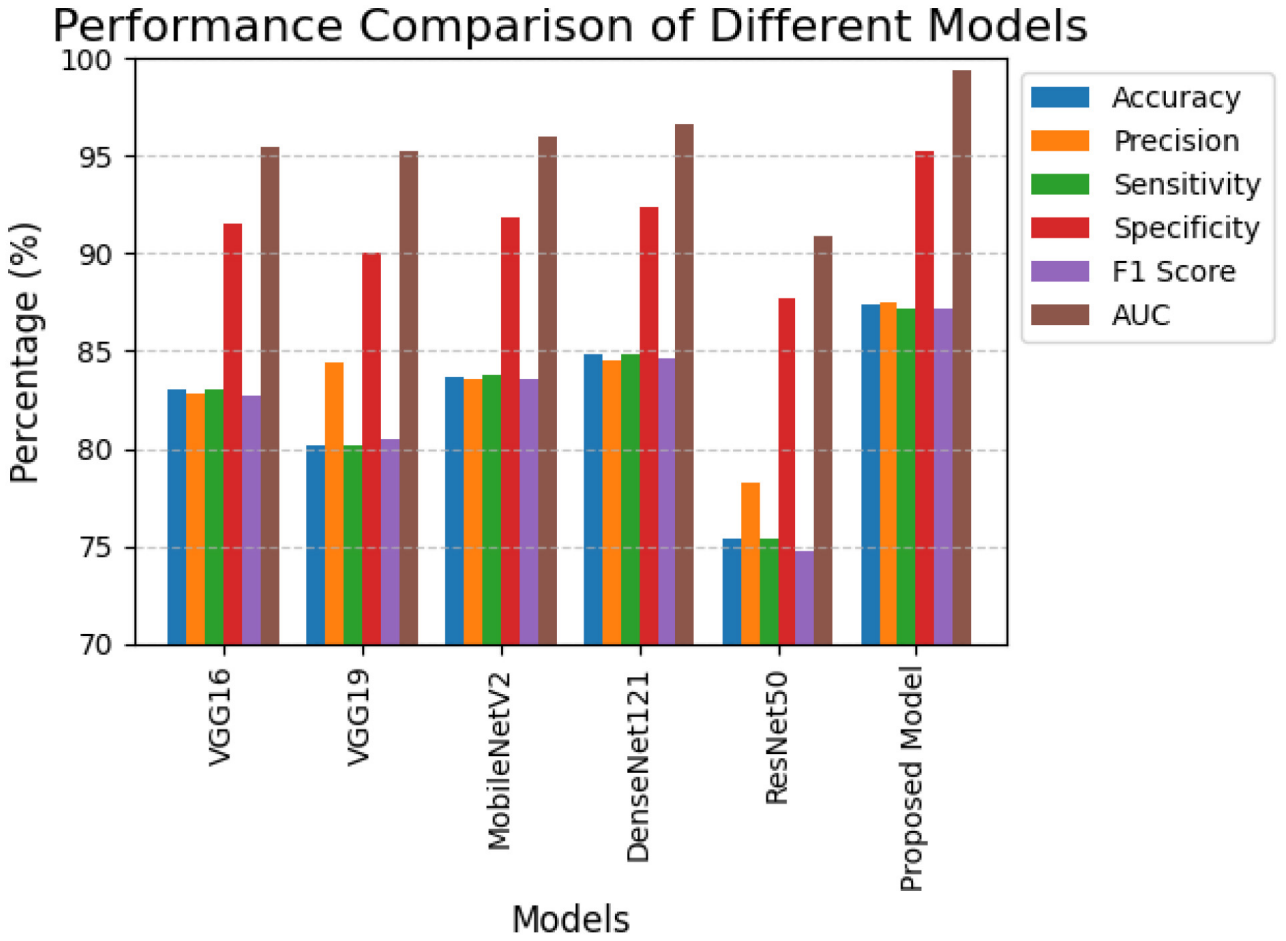
Performance comparison of different models.

The section displays all model performance metrics which include accuracy, precision, sensitivity, and specificity together with their F1 score and AUC. The KAUH-UCCTD dataset evaluates proposed model performance which researchers use to compare their model against five existing models: VGG16 and VGG19 and MobileNetV2 and DenseNet121 and ResNet50. Table 3 shows that the proposed model achieved better results than all baseline models. The system proved itself effective for uterine cancer detection because it achieved all evaluation metrics which included 87.44% accuracy and 87.48% precision and 95.20% specificity and 99.41% AUC and 87.17% F1-score. The DenseNet121 architecture among baseline models achieved second place because it reached 84.80% accuracy and 92.40% specificity which showed its ability to extract and classify complex CT image features. The VGG19 and ResNet50 models performed worse than other models because they achieved accuracies of 80.17% and 75.33% respectively. The models examined in the study show their effectiveness through the results which Figure 3 displays.

**Table 3:** Performance of the evaluated models for uterine CT image classification.

**Model**	**Accuracy**	**Precision**	**Sensitivity**	**Specificity**	**F1 Score**	**AUC**
VGG16	83.03%	82.84%	83.04%	91.52%	82.74%	95.42%
VGG19	80.17%	84.37%	80.20%	90.09%	80.50%	95.21%
MobileNetV2	83.70%	83.53%	83.76%	91.85%	83.55%	96.02%
DenseNet121	84.80%	84.54%	84.81%	92.40%	84.59%	96.62%
ResNet50	75.33%	78.25%	75.37%	87.67%	74.79%	90.87%
**Proposed model**	**87.44%**	**87.48%**	**87.13%**	**95.20%**	**87.17%**	**99.41%**

The bold values in the last row (Proposed Model) indicate the best performance results among all compared models.

The proposed model shows excellent performance because it can effectively distinguish three uterine tissue classes at all classification threshold points. The system evaluates its classification performance through a specific decision point which does not accurately represent its ability to rank results in multi-class situations with unbalanced classes. The selected threshold for classification results in misclassifications because of slight visual overlap between benign and malignant cases which leads to decreased accuracy although the system shows strong ability to differentiate between the two groups. The system shows high area under the receiver operating characteristic curve results but its actual accuracy performance remains lower than expected.

**Figure 4:**
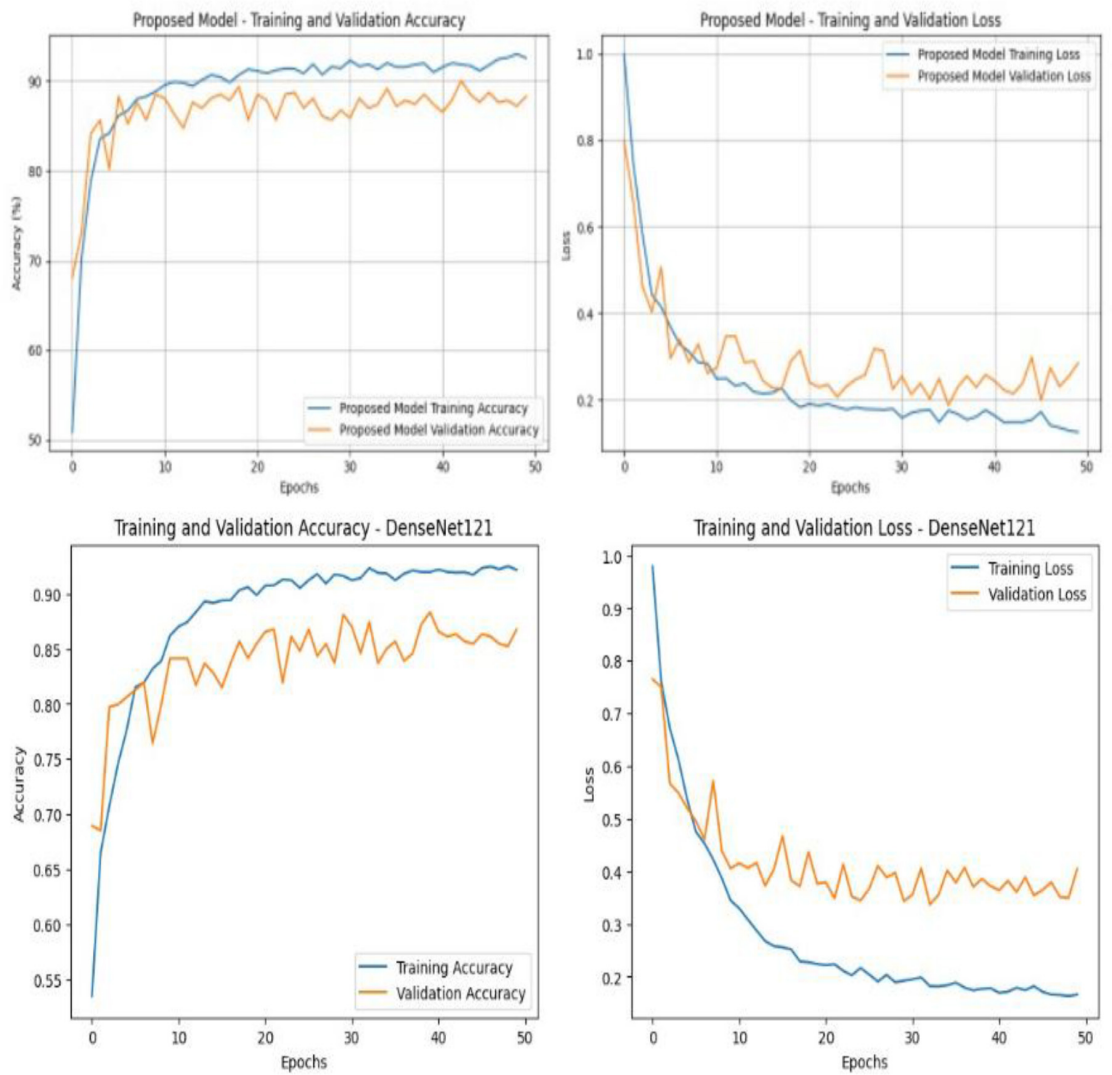
Comparison of training and validation performance between the proposed model and DenseNet121.

The results from the proposed model and the standard DenseNet121 model show their performance differences through their classification accuracy and loss measurements, which they produced during training and validation through 50 completed training epochs, as a shown Figure 4. The proposed model in the top row achieves full training accuracy through controlled training that achieves 90% accuracy while maintaining small differences between training and validation accuracy assessment. The results indicate that the model can generalize well because it achieved good results while maintaining control of overfitting problems. The proposed model's loss curve demonstrates a continuous training loss reduction while the validation loss maintains stable low levels which experience minor changes because medical CT data shows natural variability. The bottom row displays the results of DenseNet121 testing. The model achieves satisfactory training accuracy but shows poor performance during validation because it has a major gap between training results and validation outcomes. The model shows difficulties because it cannot capture all the worldwide connections that exist in images according to the high validation loss results which fail to decrease at a proper rate. The results demonstrate that using DenseNet121 with Transformer and self-attention in the proposed model has improved training stability and brought better training validation convergence results which increased generalization capabilities according to the better quantitative results that the proposed model achieved on the KAUH-UCCTD dataset.

**Figure 5:**
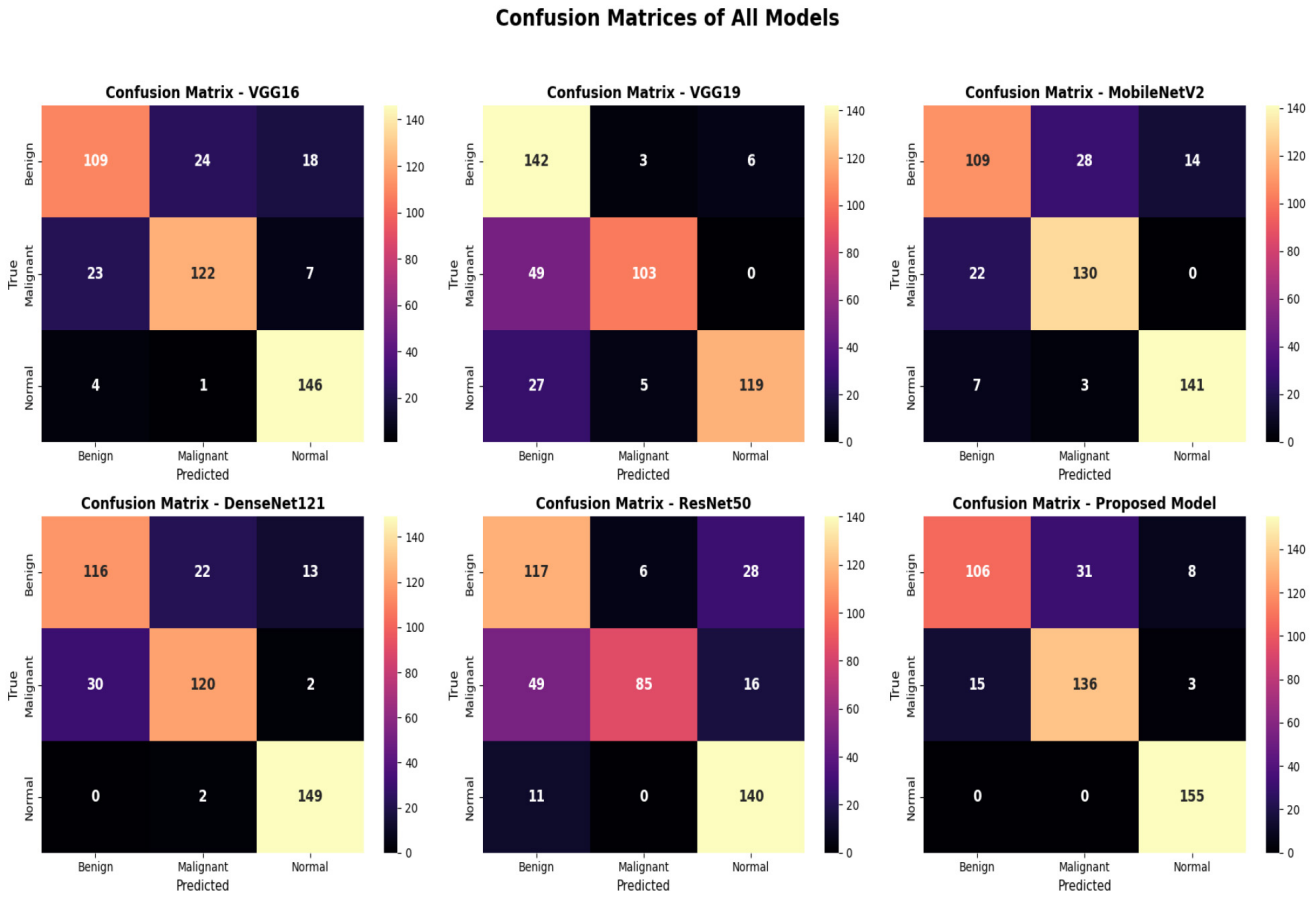
Confusion matrices of all compared models on the KAUH-UCCTD dataset.

The Figure 5 graphs below show the confusion matrices for all comparative models in classifying uterine CT images in KAUH-UCCTD in terms of VGG16, VGG19, MobileNetV2, DenseNet121, ResNet50, and our design model. The problem of identifying Benign vs. Malignant can be seen in traditional models such as VGG16 and VGG19; they are highly confused in both models, as reflected in high off-diameter points. While improvements are made in DenseNet121 and MobileNetV2 designs, but are mistaken in some cases regarding malignancies. The model presented does the best job in having the largest number of correct predictions on the main diameter, along with a considerable decrease in errors, especially in the Malignant class, exhibiting a high level of sensitivity in malign predictions and a decrease in false positives. Moreover, the model does a near-perfect job in predicting the Normal class with a negligible level of interference in the result, depicting a high level of discrimination between healthy and infected regions. The result-making matrices together ensure that the combination of the DenseNet121 model and the Self-Attention mechanism (Transformer model), in fact, increases the efficiency level in distinguishing the three classes effectively and makes the model more reliable in clinical practices.

False-negative predictions in the malignant class represent a particularly critical limitation in cancer diagnosis, as they may lead to delayed clinical intervention and adversely affect patient outcomes. Although the proposed model demonstrates high overall sensitivity and specificity, a small number of malignant cases were misclassified, primarily due to morphological similarities between benign and malignant uterine tissues. In a clinical decision-support context, such a system should be used as an assistive tool rather than a standalone diagnostic solution, with final decisions made by experienced radiologists. Moreover, future work will focus on reducing false-negative rates by incorporating cost-sensitive learning strategies, threshold optimization, or ensemble-based approaches that prioritize malignant case detection

## 5 Discussion

### 5.1 Generalization across different data sets

To ensure the validity of the results for the generalizability of the proposed model on an extended range rather than the original dataset, the performance of the model was assessed on an independent dataset, the KAUH-UCM dataset (43). The dataset consisted of 1,814 uterine MRI images, each belonging to three classes (normal, benign, and malignant). The proposed model performed satisfactorily as far as the generalization performance as it achieved an overall accuracy of 85.71%, macro-averaged precision of 85.94%, macro-averaged sensitivity of 85.25%, and macro-averaged F1 score of 85.53%. It is pertinent to note here that the model also achieved an impressive macro-averaged specificity of 92.70%. The result is significantly valuable as it substantiates the model's ability to distinguish between the negative and actual cases, which is highly desirable for an ideal medical model, as it does not produce any false positives, thus maintaining clinic relevance and reliability. The performances on the independent dataset are, however, slightly low considering the original dataset model performance, which is 88.10%.

**Figure 6:**
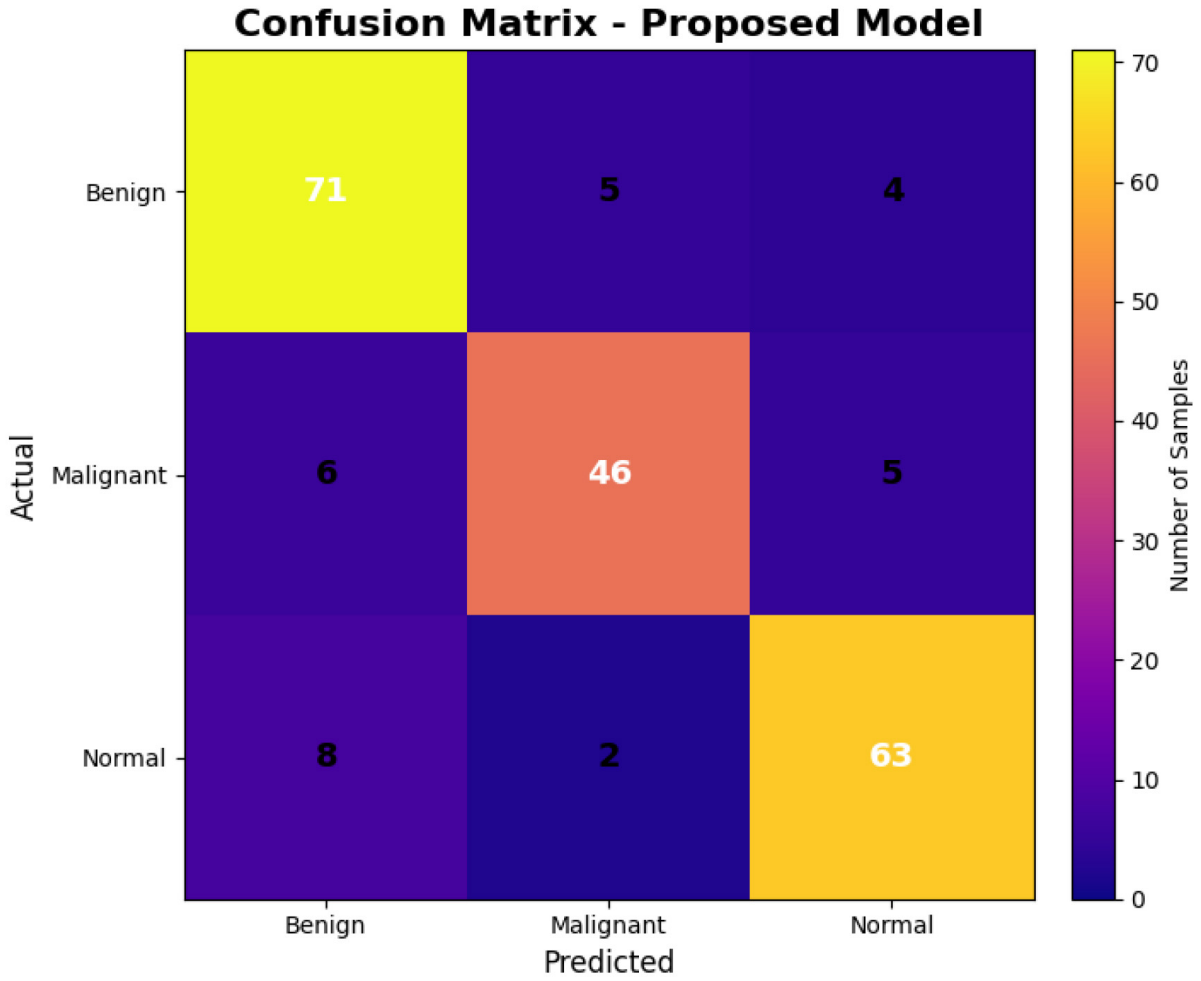
Confusion matrix of the proposed model on the KAUH-UCM Dataset.

The confusion matrix in Figure 6 represents the correctness of the classifications performed by the model for all three classes. The model was able to correctly classify a total of 71 out of 80 benign examples 88.75% accuracy, and a total of nine incorrect classifications. In the malignant class, a total of 46 out of 57 examples 80.70% accuracy were correctly classified, thus indicating the capability of the model to identify pathological abnormalities. In the normal class, the total accuracy was the highest with 63 out of 73 examples 86.30% correctly classified. The relatively mild values of the false-positive rates in the benign and malignant examples five and six, respectively suggest the existence of morphological similarities between these two classes.

### 5.2 Performance analysis and computational considerations

The model shows overall classification capabilities according to the current research study but lacks specific testing results for various tumor stages and sizes and different body parts. The model testing process needs to include subgroup testing because it helps determine how well the model works in clinical settings across various patient groups. The study failed to evaluate both computational efficiency and inference time which count as two essential requirements for real-time clinical use. The proposed model uses both a DenseNet121 backbone and a lightweight transformer encoder which helps to achieve fast inference times but the research team plans to study runtime performance through detailed analysis and optimization work. The future research will evaluate tumor subtypes and anatomical variations through detailed performance tests while also working to enhance model inference speed for actual clinical application.

### 5.3 Practical and research implications

The proposed deep learning algorithm would integrate well with the medical system of the hospital as a clinical decision-support system for radiologists for early and accurate diagnosis of uterine cancer using CT scanning images of the uterus. The integration process would begin with the acquisition of the CT scan images by imaging technology available at the hospital, which would all be stored in the Picture Archiving and Communication System (PACS) of the hospital. These would then ideally flow automatically to the proposed system for resizing and normalization according to the training environment requirements. The DenseNet121-Transformer deep learning system would then process these images and produce probabilities for all three types: normal, benign, and malignant uteri. The output would then ideally flow back to the hospital information system (HIS) and radiology information system (RIS), and the radiologist would easily access the output of the system by means of an interactive user interface without having to actually undergo all the processes of the machine learning system since it acts as a decision support tool for radiologists and doctors of the hospital.

### 5.4 Limitations of the study

Although there are promising outcomes observed with the proposed model regarding the (DenseNet121-Transformer) architecture, there are a few shortcomings and areas that need to be addressed in the future for improvement. Although there are multiple views available in the data set presented (axial, sagittal, and coronal), it is still observed that the model can independently analyze these different views without taking into consideration the other data available in between the slices or in 3D. As observed in the data set, it contains a certain level of class imbalance, which can affect the training process, even when data augmentation strategies are used. Although there is an improvement in explainability on a feature level through the attention mechanism in the Transformer, there are no techniques in this study presented that focus on the use of other explainability tools, which are essential in increasing trust. Although the proposed model demonstrates consistent performance improvements over the comparative methods across all evaluation metrics, statistical significance analysis was not performed in this study. The current research work exhibits a specific limitation here. The upcoming research will use formal statistical testing through multiple experimental repetitions to establish the strength and dependability of the suggested method.

The study conducted their study at one complete tertiary healthcare facility which delivered superior imaging services and treated patients from diverse backgrounds. The research achieves dependable results for model development and testing yet remains restricted to one research site. The upcoming research will use datasets from multiple medical facilities and different equipment manufacturers to improve the model's performance in various clinical environments.

## 6 Conclusion and future works

This study presented an efficient hybrid deep learning architecture that was demonstrated, comprising the combination of DenseNet121 and the self-attention mechanism based on the Transformer for the classification of uterine CT images into normal, benign, and malignant images based on the KAUH-UCCTD dataset. In the study, the proposed hybrid model efficiently combined the strength of convolutional neural networks in identifying local textual features in the images along with the ability of the Transformer encoder to handle the images globally. The results obtained from the experiment have shown that there is superiority in the proposed method over different advanced methods such as VGG16, VGG19, MobileNetV2, DenseNet121, and ResNet50 based on different parameters such as accuracy, sensitivity, specificity, F1-score, and AUC.

Future work will be done by extending the study to validate the proposed framework on multi-institutional multi-center datasets, which improves the generalizability and clinical reliability of the framework. The inclusion of 3D volumetric information and interslice relationships from CT examinations could lead to even better diagnostic performance. The integration of XAI techniques, such as attention maps or visualization saliency, would make the model more interpretable and increase clinician trust in the models. Other future works may involve real-time deployment in hospital environments, optimization for computational efficiency, and integration with clinical workflows for practical decision-making during routine medical practice.

## Data Availability

The original contributions presented in the study are included in the article/supplementary material, further inquiries can be directed to the corresponding author.

## References

[1] NCI Division of Cancer Control and Population Sciences. Cancer Stat Facts: Uterine Cancer. Available online at: https://seer.cancer.gov/statfacts/html/corp.html (Accessed December 25, 2024).

[2] Felix AS Brinton LA. Cancer progress and priorities: uterine cancer. Cancer Epidemiol Biomarkers Prev. (2018) 27:985–94. doi: 10.1158/1055-9965.EPI-18-0264 30181320 PMC6504985

[3] Stewart EA Cookson CL Gandolfo RA Schulze-Rath R. Epidemiology of uterine fibroids: a systematic review. BJOG. (2017) 124:1501–12. doi: 10.1111/1471-0528.14640 28296146

[4] Ledford LR Lockwood S. Scope and epidemiology of gynecologic cancers: an overview. in Seminars in Oncology Nursing. Amsterdam: Elsevier (2019). p. 147–50. doi: 10.1016/j.soncn.2019.03.002 30902519

[5] Venkatesh A Isaacs C. Trends in Uterine cancer mortality in the United States: a 50-year population-based analysis. Obstet Gynecol. (2024) 143:e130–1. doi: 10.1097/AOG.0000000000005543 38513250

[6] Somasegar S Bashi A Lang SM Liao CI Johnson C Darcy KM . Trends in uterine cancer mortality in the United States: a 50-year population-based analysis. Obstet Gynecol. (2023) 142:978–86. doi: 10.1097/AOG.0000000000005321 37678887 PMC10510793

[7] Henley SJ. Uterine cancer incidence and mortality—United States, 1999–2016. MMWR Morb Mortal Wkly Rep. (2018) 67:1333–8. doi: 10.15585/mmwr.mm6748a1 30521505 PMC6329484

[8] Yu H Luo S Ji J Wang Z Zhi W Mo N . A deep-learning-based artificial intelligence system for the pathology diagnosis of uterine smooth muscle tumor. Life. (2022) 13:3. doi: 10.3390/life13010003 36675952 PMC9864148

[9] Jan YT Tsai PS Huang WH Chou LY Huang SC Wang JZ . Machine learning combined with radiomics and deep learning features extracted from CT images: a novel AI model to distinguish benign from malignant ovarian tumors. Insights Imaging. (2023) 14:68. doi: 10.1186/s13244-023-01412-x 37093321 PMC10126170

[10] Mao W Chen C Gao H Xiong L Lin Y. A deep learning-based automatic staging method for early endometrial cancer on MRI images. Front. Physiol. (2022) 13:974245. doi: 10.3389/fphys.2022.974245 36111158 PMC9468895

[11] Buddenkotte T Rundo L Woitek R Sanchez LE Beer L Crispin-Ortuzar M . Deep learning-based segmentation of multisite disease in ovarian cancer. Eur Radiol Exp. (2023) 7:77. doi: 10.1186/s41747-023-00388-z 38057616 PMC10700248

[12] Zhu Z Xu S Li K Zhao W Xu T Xia Y . Abnormal uterine classification based on an improved YOLOv5 framework from ultrasound images. in Sixteenth International Conference on Graphics and Image Processing (ICGIP 2024) (2025) p. 256–65. doi: 10.1117/12.3060403

[13] Sepehr J Caprio A Sam L Lee BC Sabuncu MR Lamparello NA. et al. Predicting clinical outcomes and symptom relief in uterine fibroid embolization using machine learning on MRI features. AI. 6:200. doi: 10.3390/ai6090200

[14] Tinelli A Morciano A Sparic R Hatirnaz S Malgieri LE Malvasi A . Artificial intelligence and uterine fibroids: a useful combination for diagnosis and treatment. J Clin Med. 14:3454. doi: 10.3390/jcm14103454 40429449 PMC12112542

[15] Öz I Yegin EE Öz AU Ulukaya E. An AI-driven clinical decision support framework utilizing female sex hormone parameters for surgical decision guidance in uterine fibroid management. Medicina (B Aires). (2025) 62:1. doi: 10.3390/medicina62010001 41597287 PMC12842902

[16] Xu B Liu J Fang M Zhu H Zhang Y Zhang H . Multicenter deep learning-based automatic delineation of CTV and PTV in uterine malignancy CT imaging. Radiother Oncol. (2025) 214:111212. doi: 10.1016/j.radonc.2025.111212 41120056

[17] Wang T Wen Y Wang Z. nnU-Net based segmentation and 3D reconstruction of uterine fibroids with MRI images for HIFU surgery planning. BMC Med. Imaging. (2024) 24:233. doi: 10.1186/s12880-024-01385-3 39243001 PMC11380377

[18] Li C He Z Lv F Liao H Xiao Z. Predicting the prognosis of HIFU ablation of uterine fibroids using a deep learning-Based 3D super-resolution DWI radiomics model: a multicenter Study. Acad. Radiol (2024) 31:4996–5007. doi: 10.1016/j.acra.2024.06.027 38969576

[19] Göker H. Detection of cervical cancer from uterine cervix images using transfer learning architectures. Eskisehir Tech Univ J Sci Technol A Appl Sci Eng. 25:222–39. doi: 10.18038/estubtda.1384489

[20] Santoro M Zybin V Coada VA Mantovani G Paolani G Stanislao MD . Machine learning applied to pre-operative computed-tomography-based radiomic features can accurately differentiate uterine leiomyoma from leiomyosarcoma: a pilot study. Cancers (Basel). (2024) 16:1570. doi: 10.3390/cancers16081570 38672651 PMC11048510

[21] Xi H Wang W. Deep learning based uterine fibroid detection in ultrasound images. BMC Med Imaging. (2014) 24:218. doi: 10.1186/s12880-024-01389-z 39160500 PMC11331772

[22] Alswilem L Pacal M. Computational efficiency and accuracy of deep learning models for automated breast cancer detection in ultrasound imaging. Artif Intell Appl Sci. (2025) 1:1–6. doi: 10.69882/adba.ai.2025071

[23] Alpsalaz SD Aslan E Özüpak Y Alpsalaz F Uzel H Bereznychenko V . Hybrid deep learning with attention fusion for enhanced colon cancer detection. Sci. Rep. (2025) 15:45583. doi: 10.1038/s41598-025-29447-8 PMC12754056 41315603

[24] Çakmak Y. Machine learning approaches for enhanced diagnosis of hematological disorders. Comput Syst Artif Intell. (2015) 1:8–14. doi: 10.69882/adba.csai.2025072

[25] Çakmak Y Pacal N. Deep learning for automated breast cancer detection in ultrasound: A comparative study of four CNN architectures. Artif Intell Appl Sci. (2025) 1:13–9. doi: 10.69882/adba.ai.2025073

[26] Aslan E Alpsalaz SD Alpsalaz F Uzel H. Alzheimer's classification with a MaxViT-based deep learning model using magnetic resonance imaging. J Appl Sci TechnolTrends. (2025) 6: doi: 10.38094/jastt62453

[27] Kör H Mazman R. Brain tumor detection and classification with deep learning based CNN method. Comput Syst Artif Intell.(2025) 1:15–9. doi: 10.69882/adba.csai.2025073

[28] Kiran HE. Deep learning-based detection of abdominal diseases using YOLOv9 models and advanced pre-processing techniques. Comput Electron Med. 2:20–5. doi: 10.69882/adba.cem.2025014

[29] Abushahla KH Pala MA. Optimizing diabetes prediction: addressing data imbalance with machine learning algorithms. ADBA Comput Sci. (2024) 1:26–35. doi: 10.69882/adba.cs.2024075

[30] Rama J Nalini C Kumaravel A. Image pre-processing: enhance the performance of medical image classification using various data augmentation technique. ACCENTS Trans Image Process Comput Vis. (2019) 5:14–17. doi: 10.19101/TIPCV.413001

[31] Goceri E. Medical image data augmentation: techniques, comparisons and interpretations. Artif. Intell Rev. (2023) 56:12561–605. doi: 10.1007/s10462-023-10453-z 37362888 PMC10027281

[32] Siddarth SG Chokkalingam S. DenseNet 121 framework for automatic feature extraction of diabetic retinopathy images. In International Conference on Emerging Systems and Intelligent Computing(ESIC); 2024 Feb; New York, NY: IEEE, (2024), p. 338–42. doi: 10.1109/ESIC60604.2024.10481664

[33] Rajkumar R. Deep learning feature extraction using attention-based DenseNet 121 for copy move forgery detection. Int. J. Image Graph. (2023) 23:2350042. doi: 10.1142/S0219467823500420

[34] Liu Y Sun G Qiu Y Zhang L Chhatkuli A Van Gool L . Transformer in convolutional neural networks. arXiv [Preprint]. arXiv:2106.03180 (2021).

[35] Dosovitskiy A. An image is worth 16x16 words: transformers for image recognition at scale. arXiv [Preprint] *arXiv:11929* (2020).

[36] Wu H Xiao B Codella N Liu M Dai X Yuan L . Cvt: introducing convolutions to vision transformers. in Proceedings of the IEEE/CVF international conference on computer vision. (2021) 22–31. doi: 10.1109/ICCV48922.2021.00009

[37] Xiao X Zhang D Hu G Jiang Y Xia S. CNN–MHSA: a Convolutional neural network and multi-head self-attention combined approach for detecting phishing websites. Neural Netw. (2020) 125:303–312. doi: 10.1016/j.neunet.2020.02.013 32172140

[38] Tan H Liu X Yin B Li X. MHSA-Net: multihead self-attention network for occluded person re-identification. IEEE Trans Neural Netw Learn Syst. (2022) 34:8210–24. doi: 10.1109/TNNLS.2022.3144163 35312622

[39] Raghu S Sriraam N. Optimal configuration of multilayer perceptron neural network classifier for recognition of intracranial epileptic seizures. Expert Syst Appl. (2017) 89:205–21. doi: 10.1016/j.eswa.2017.07.029

[40] Dino HI Abdulrazzaq MB. Facial expression classification based on SVM, KNN and MLP classifiers. 2019 In: *International Conference on Advanced Science and Engineering (ICOASE)*, IEEE (2019) 70–5. doi: 10.1109/ICOASE.2019.8723728

[41] Müller D Soto-Rey I Kramer F. Towards a guideline for evaluation metrics in medical image segmentation. BMC Res Notes. (2022). 15:1210. doi: 10.1186/s13104-022-06096-y 35725483 PMC9208116

[42] Heydarian M Doyle TE Samavi R. MLCM: multi-label confusion matrix. Ieee Access (2022). 10:19083–95. doi: 10.1109/ACCESS.2022.3151048

[43] Altal OF Sindiani AM Mhannaa HYA Alhatamleh S Amin M Akhdar HF . WOAENet: a whale optimization-guided ensemble deep learning with soft voting for uterine cancer diagnosis based on MRI images. Front Artif Intell. (2025) 8:1664201. 41190038 10.3389/frai.2025.1664201 PMC12580201

